# Calcification Formation for Development, Defense, and Repair of the Human Body?

**DOI:** 10.3390/jcm13195691

**Published:** 2024-09-25

**Authors:** Pim A. de Jong, Daniel Bos, W. P. Th. M. Mali

**Affiliations:** 1 Department of Radiology, University Medical Center Utrecht, Utrecht University, 3584 CS Utrecht, The Netherlands; w.mali@umcutrecht.nl; 2 Department of Epidemiology, Erasmus Medical Center, 3015 GD Rotterdam, The Netherlands; d.bos@erasmusmc.nl; 3 Department of Radiology and Nuclear Medicine, Erasmus Medical Center, 3015 GD Rotterdam, The Netherlands; 4 Department of Epidemiology, Harvard T.H. Chan School of Public Health, Boston, MA 02138, USA

## 1. The Process of Extracellular Calcification

**Figure 1:**
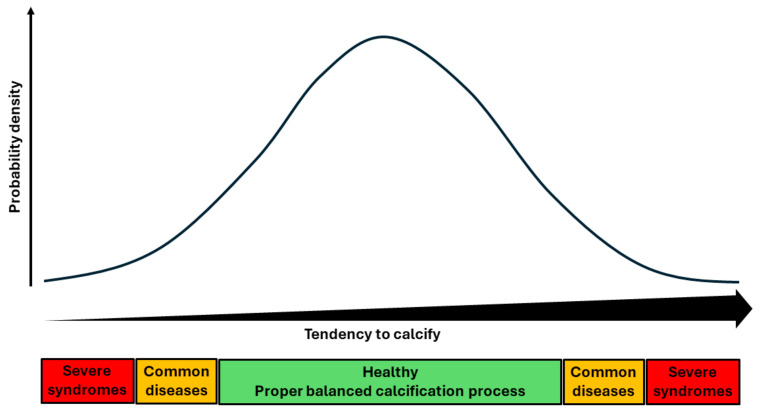
Distribution of calcification formation in the human body.

Calcium deposits commonly occur in the human body in any type of tissue through an actively regulated process [1]. First and foremost, extracellular calcification is responsible for a balanced build-up and homeostasis of the skeleton and rapid fracture healing, which is essential for mobility and survival. We and others propose that bone formation-like processes are also crucial for an adequate defense against certain infections [2,3]. More controversial is the hypothesis that bone formation-like processes are utilized for the repair of arteries that are damaged by atherosclerotic plaques and elastin fractures [4,5,6,7,8]. The calcification process is a basic mechanism influenced by many genes and environmental factors, all of which may vary [9]. Hence, calcification in the population may be present in a broad range of phenotypes, which can be visualized as a bell-shaped distribution (Figure 1) [10]. Inherent to evolution, most of the benefits of the calcification process are to be found around the center of the distribution, whilst potential harmful effects of the process may be found in the tails of the distribution. Against this background, it makes sense that the majority of the population resides around the middle of this distribution, which can be translated to the fact that most people feature well-developed bones and fracture repair mechanisms, can survive an infection, and are able to calcify atherosclerotic plaques and elastin fractures fast enough. In the tails of the distribution, however, the ability to ‘calcify’ is too limited or too extensive. On the left side of the distribution, this can result in an inability to control infections such as mycobacteria tuberculosis and parasites; multiple fractures or poor fracture healing; aneurysm formation; and longer periods of atherosclerotic plaque instability, resulting in myocardial infarction or stroke. Conversely, on the right side of the distribution, people can develop sclerotic fragile bones, stiffness and pain from osteoarthritis, and arterial stiffening with subsequent vascular disease.

In order to provide evidence for this hypothesis around the calcification process (Figure 1), major challenges have to be overcome. First, it is extremely difficult to prove the existence of a limited ‘ability to calcify’, since, contrary to visualizing extensive calcifications with imaging, there are no adequate tools to visualize this. Second, it is difficult to judge the ‘strength’ of the calcification process since the degree of calcification is also dependent on the difficult-to-quantify degree of abnormal development, insufficient defense against infections, or the amount of arterial damage. Third, chronic diseases (for example, due to stiff vessels) require a long observation time and are more difficult to investigate compared to acute diseases. Fourth, at least later in life, opposing effects are observed in epidemiological studies that investigate the calcification process in arteries and skeleton. For example, the osteoporosis paradox is well known, where more vascular calcification is associated with lower bone density. It is possible that the poor vasculature is a cause of rapid bone loss, but truly opposite effects may also be present in this pleiotropic system. Fifth, at early stages, microcalcifications in arterial plaque are thought to destabilize plaques. We believe that this is consistent with our viewpoint, as people at the lower ends of the spectrum will experience longer periods with microcalcifications.

Despite these major challenges, fragmented evidence from the fields of infections, bone disease, and arterial disease is available and will be summarized in the following paragraphs to strengthen our hypothesis.

## 2. Calcification as a Defense Mechanism against Infections

To comprehend the significance of calcification in the context of infectious diseases, it is imperative to delve into historical perspectives. Following the initial epidemiological transition roughly 10,000 years ago, wherein human populations transitioned from nomadic to agrarian lifestyles, the average lifespan was approximately 35 years [11]. This age was predominantly influenced by mortality stemming from infectious diseases, which constituted a strong and widespread selective pressure on the human genome. Through vaccination, enhanced hygiene practices, and antibiotics, infectious diseases were effectively prevented or treated, marking the second epidemiological transition around the year 1800, thereby extending life expectancy to around 60 years. An inherent mechanism employed by the human body to combat infections is calcification. Presently, this defensive strategy can still be observed, such as during the calcific encapsulation of parasites and the formation of primary complexes attributed to Mycobacterium tuberculosis [2,3]. Calcification may be considered to be a final line of defense against chronic infections or can be seen as tolerance against a microorganism, particularly when the physiological cost of eradication outweighs the benefits to the organism [10].

## 3. Infections, Longer Survival, and Higher Arterial Calcification Burden

Bioarcheological studies from before the second epidemiologic transition can provide us with the best information about the selection pressure exerted by infections since people with a weak defense died (left side of the distribution in Figure 1), although the data are cross-sectional and at risk of selection bias. Most evidence of the complex interrelation between infection, the strength of the calcification formation, and survival comes from two larger bioarcheological cohorts from before the second epidemiologic transition. The first cohort is the Swedisch Blackfrairs study [12]. In this study, 290 human skeletal remains from brothers of the Blackfriars convent as well as wealthy individuals were excavated. They were buried between the years 1237 and 1536. Through the careful analysis of the content of the skulls, the investigators found fragile calcified cylinder-shaped fragments measuring 5 mm in diameter, which represent severe carotid siphon calcification. Twenty-five skulls contained these calcifications, pointing at a strong calcification mechanism at the right side of the distribution. Of the 153 people who died between 20 and 39 years, none had calcifications; of the 97 who died between 40 and 59 years, 11 (11.3%) had calcifications; while in the 40 who died after the age of 60, 14 (35%) had calcifications. Interestingly, the cohort was also investigated for the occurrence of infectious diseases such as septic arthritis, osteomyelitis, periostitis, and specific infections such as leprosy, tuberculosis, and syphilis. These infectious diseases were seen in just 1 of the 25 (4%) people with calcifications, while they were much more common in those without calcifications. This cohort provides some evidence that the strong calcification process was related to fewer infections and better survival.

The second cohort is the Horus study in which 137 Egyptian and non-Egyptian mummies who had lived over a 4000-year period in four different ancient cultures were imaged with computed tomography [13]. The mean age of these mummies was 36 years. In 34% of the mummies, calcifications were found to varying degrees in up to five arterial beds, The mean age at death of mummies without calcification, with 1–2 beds calcified, and with 3–5 beds calcified was 32, 42, and 44 years, respectively.

So, both cohorts found that a strong calcification process in the arteries was associated with older age at death. This is explained currently by the dictum that aging goes with vascular calcification. However, in an era where infectious diseases were the dominant cause of death, another explanation could be that a stronger calcification mechanism is also responsible for a better defense against infections and improved survival. Yet, since the studies are cross-sectional, cause and effect cannot be determined.

## 4. Calcification Formation for Development and Maintenance of the Skeleton

Obviously, proper mineralization leading to a strong skeleton and the rapid healing of fractures is essential for survival. When the strength of the calcification process in the population is distributed as in Figure 1, most people are around optimal bone strength, but some have relatively weak bones due to insufficient calcification and others have stiff bones due to calcification that is too strong. An accepted measure for the degree of calcification of the bone is bone mineral density (BMD). One should expect that when the strength of the calcification process is distributed in the population according to Figure 1, the BMD/mortality–morbidity distribution is U-shaped. A recent study using advanced data analysis indeed found that in males, a U-shaped BMD/mortality distribution was present [14].

Numerous studies have focused on the left side where the important clinical entity of osteoporosis is located. Several meta-analyses have shown that low BMD is associated with an increased risk for the fractures of the wrist, hip, and spine [15]. A low BMD is also related to a higher all-cause and cardiovascular mortality, while recently, it was shown to be a risk factor for infections and sepsis [16].

On the right side of the distribution, a high BMD is found. Although less knowledge is available, patients with osteoarthritis have higher bone mineral density. Severely calcified bone is less flexible and stiffer, which can lead to joint damage, which, in turn, is repaired with osteophytosis [17]. Osteophyte size and presence are then again determined by our distribution. Clinical studies about high BMD and mortality and morbidity are lacking; however, in the rare disease osteopetrosis, excessive bone formation results in significant morbidity and early mortality [18]. Another rare disease is the excessive calcifications of joints and arteries (CALJA) [19]. These patients develop severe arterial calcifications, arthritis, osteophytes, and soft tissue calcification around joints.

In summary, osteoporosis and osteoarthritis are a different side of the same coin and together they cover both sides of the distribution [20].

## 5. Calcification Formation for Maintenance and Damage Repair in Arteries

Traditionally, within the medical field, arterial calcification has been perceived as the inert final stage of atherosclerotic disease. However, currently, arterial calcification is seen as an active metabolic process susceptible to modulation. While certain publications have posited arterial calcification as a reparative mechanism, widespread acceptance of this concept has not yet been attained [4,5,6,7,8]. We propose that arterial calcification serves as a repair process for damage affecting the tunica intima, elastic lamina, or tunica media, with various triggers and outcomes. The damage to the endothelium and elastin fibers, however, share a common phenotypic expression in the form of arterial calcification. The mechanisms by which the arterial wall sustains damage can vary significantly, encompassing hereditary, constitutional, metabolic, endocrine, environmental, radiation-induced, or biomechanical factors stemming from pulse pressure. The degree of arterial calcification is, thus, contingent upon two key factors: the severity of the damage incurred and the robustness of the repair mechanisms. Near the mean, the strength of the calcification process typically proves beneficial for most individuals (Figure 1). In advanced ages, arterial calcification becomes prevalent among the population; however, the majority of individuals do not succumb to or suffer from clinical vascular diseases. In these individuals, the arterial repair process seems neither deficient nor excessive.

On the left side of the distribution, according to our theory, we expect that insufficient calcification would lead to aneurysms, dissections, and plaque rupture in arteries in the population. Most studies, unfortunately, only measure calcium scores, but angiographic techniques are needed to correct the cumulative amount of arterial damage represented in the total plaque burden. If this correction is carried out, it is indeed consistently observed that low calcification proportion is associated with acute cardiovascular diseases. For example, in the Scot-Heart trial, it was shown that an increased burden of low attenuation noncalcified plaque is the principal predictor of coronary events above and beyond the other established risk factors including coronary artery stenosis severity [21]. Several other studies have shown that patients with stable in comparison to unstable angina are more likely to have calcified plaques [22,23,24,25]. Also, patients with only noncalcified coronary atherosclerosis have a high risk of events when compared with people with a similar plaque burden that is calcified. However, most studies only report on a coronary calcium zero group, which is strongly diluted with persons without atherosclerotic coronary damage [26], and so strongly diluting the risk of noncalcified plaques. In abdominal aorta and intracranial aneurysms, it is known that calcified aneurysms grow slower, and it is thought that calcified plaques rupture less, suggesting that the calcific repair mechanism was able to prevent events [27,28,29,30,31,32].

On the right side of the distribution, we expect that either too much repair activity or too much damage would lead to severe arterial calcification. Investigations into the effect of the severity of the calcifications on the long-term outcome showed that in all the investigated vascular territories, severe calcifications were always related to the worst outcome [33,34,35,36,37]. There are several explanations. First, severe intima damage, which results in severe atherosclerosis, calcifies in those with a normal or extreme calcific response. Second, tunica media and internal elastic lamina damage can result in annular calcification. Annular calcifications limit compensatory dilatation and outward remodeling; lack the trigger to form collateral vessels; and cause vascular stiffening and limit the windkessel function. These physiological effects have been mainly studied in the (abdominal) aorta. Here, the pulse wave velocity has been accepted as a measure of the stiffness of the aorta, and this stiffness was strongly related to calcific lesions. Both abdominal aortic calcification and pulse wave velocity are strongly related to multiple chronic vascular diseases such as dementia, heart failure, kidney dysfunction, liver fibrosis, pre-eclampsia, and testicular atrophy [38]. More recently, also in the intracranial internal carotid artery (the carotid siphon) and in the femoral artery, annular calcifications were most strongly related to stroke, cognitive decline, and amputation, respectively [39,40].

On the far-right side of the distribution, multiple rare diseases are associated with excessive calcification formation in arteries, severe phenotypes, and premature mortality. The diseases include generalized arterial calcification of infancy, pseudoxanthoma elasticum, progeria, and idiopathic basal ganglia calcification [41,42,43]. Usually, in these syndromes, calcification-inhibiting pathways are compromised. The diseases are characterized by severe symptoms such as blindness, dementia, parkinsonism, and vascular events such as myocardial infarction or stroke. It is most likely that the pathophysiological pathway to cardiovascular events is through arterial stiffening.

## 6. Consequences for Diagnosis and Treatment of Cardiovascular Disease

In this paragraph, we will focus on the implications of our viewpoint for cardiovascular medicine. The main consequence is that we do not only focus on the atherosclerotic or mechanical damage but also on the unbalanced repair mechanism. As we will demonstrate, adopting this perspective on calcification formation can significantly influence the diagnostic process, prevention, and treatment of both acute and chronic cardiovascular diseases.

Firstly, there are diagnostic implications. The view that arterial calcification primarily serves a beneficial role implies that, for the majority of individuals, arterial calcifications represent a normal reparative mechanism that does not necessarily lead to clinical vascular diseases. Unfortunately, there is no test to measure the setpoint of the calcific response. Therefore, we propose a much broader role for wall imaging. Zero, low, and intermediate calcium scores are only reassuring when in balance with the cumulative amount of damage, and thus, when the burden of noncalcified plaque is limited. The assessment of the amount of noncalcified plaque requires more advanced wall imaging. Severe and annular arterial calcifications are likely always concerning, and in such cases, the diagnostic implication may involve a more thorough assessment of collateral status. This could be particularly relevant as vascular interventions may be more beneficial in individuals with poor collateral circulation [44].

Secondly, there could be consequences for treatment. Of course, the prevention of vascular damage is paramount, but, as soon as damage has occurred, research should show whether the calcification repair mechanism could be left as is, or if it should be stimulated or inhibited. In such a research setting, individuals with significant damage or with a high propensity to calcify could be treated with anti-calcifying medication to stop or regress the calcification process. This strategy has a history in medicine, but it was apparently forgotten [45,46,47]. Recently, it has been gaining more and more attention, especially for rare calcific diseases [48,49]. In the Netherlands, the drug Etidronate is prescribed to patients with moderate to severe arterial calcifications with PXE. In patients with a low propensity to calcify and with unstable plaques or aneurysms, it can be tested whether during a short period, the calcification process should be stimulated.

## 7. How to Acquire Further Evidence?

First, although cross-sectional in nature, further studies in mummies, animals, and animal fossil records are of interest [50]. In mummies, age- and sex-standardized scores of bone mineral density (showing the location on the distribution in Figure 1) could be related to vascular calcifications, osteoarthritis, hallmarks of infections, and survival. Similar investigations could be conducted in the fossil record as fossils that are millions of years old are preserved in museums around the world.

Second, experiments with calcification-modifying medication in humans with rare diseases could provide further proof. These are feasible for the right end of the spectrum, and randomized trials of anti-calcific drugs in rare (neuro)vascular syndromes are ongoing. In these trials, it is essential to monitor both acute and chronic vascular problems, the joints, and the skeleton to gain knowledge on the bone–vascular interaction.

Third, in common cardiovascular diseases, trials of pro-calcific agents in patients with noncalcified plaque or aneurysms could test whether such treatment indeed leads to faster calcification, a shorter period of instability, and subsequently, less myocardial infarction, coronary stenosis, aneurysm rupture, and better outcome.

Fourth, in the population on the right end of the spectrum, trials could test whether the onset of organ failure including dementia could be delayed by decalcifying agents. In such trials, it would be important to manage aneurysms and noncalcified plaques properly.

## 8. Conclusions

The process of extracellular calcification in the human body should be seen as beneficial in the development and homeostasis of bone, defense against certain infections, and in repairing vascular damage. Due to this common calcification mechanism, these processes are related to each other. When the calcification mechanism is in imbalance, it can cause disease and can affect all three processes. We have illustrated how adopting this perspective holds substantial implications for the diagnosis and treatment of cardiovascular diseases.
